# Reoperation frequency after transverse preputial Island flap urethroplasty “Duckett’s technique” in treatment of severe hypospadias: A single center study

**DOI:** 10.3389/fped.2022.1030649

**Published:** 2023-01-09

**Authors:** Jiayi Li, Pei Liu, Zhenzhen Yang, Xinyu Wang, Songqiao Fan, Zonghan Li, Hongcheng Song, Weiping Zhang

**Affiliations:** Department of Urology, Beijing Children’s Hospital, Capital Medical University, National Center for Children’s Health, Beijing, China

**Keywords:** hypospadias, complication, transverse preputial island flap, operation, chilren

## Abstract

**Purpose:**

Transverse Preputial Island Flap Urethroplasty (TPIFU) is one of the most common techniques for treating severe hypospadias. Studies on the reoperation frequency after TPIFU is lacking. In the present study, we reported our clinical outcomes of severe hypospadias treated with one-staged TPIFU and analyzed the operation frequency.

**Methods:**

We retrospectively analyzed the clinical data of severe hypospadias patients who underwent one-stage TPIFU from December 2018 to December 2019 in the department of Urology at Beijing Children's Hospital. A stepwise approach was used to manage the curvature. Severe hypospadias was defined as those residual curvature was higher than 30° after degloving. Urethroplasty complications included fistula, urethral stricture, and diverticulum. The short-term cure was identified as no complications occurring for 12 months after the date of last-time surgery. The reoperation rate and operation frequency of TPIFU were analyzed.

**Results:**

A total of 136 patients who underwent one-stage TPIFU were included in the study. The follow-up after primary urethroplasty ranged from 22 to 50 months. The median age at primary surgery was 22.5 months (range from 13 to 132 months). After primary TPIFU surgery, 53 (39%) patients underwent additional surgical interventions to treat postoperative complications. Among them, 24 patients (17.6%) developed fistula, 17 patients (12.5%) developed urethral stricture and 11 patients (8.1%) developed diverticulum. After the second surgery, five patients remained fistula, five patients remained urethral stricture, and seven patients remained diverticulum. Overall, 61% (85 patients) met the cured standard after the primary operation, and the two operations cure rate was 87.5% (119 patients). 91.2% (124 patients) were cured in three operations.

**Conclusions:**

Although the complication rates after primary TPIFU were relatively high, more than half of patients achieved short-term cured through a single operation, and the cure rate after two or three operations was acceptable.

## Introduction

Hypospadias is one of the most common congenital malformations of boys, of which proximal hypospadias account for 20% of all cases (1). Despite the evaluation and development of surgical techniques, the complication rate and reoperation rate of proximal hypospadias repair are still far from satisfactory (2). For proximal hypospadias with severe curvature, transection of the urethral plate is inevitable to correct ventral curvature (VC), and the simultaneous urethral plate substitution must be performed with graft implantation. In this situation, some surgeons prefer single-stage urethroplasty, such as transverse preputial island flap urethroplasty (TPIFU), the Koyanagi technique, whereas others advocate two-stage repair to achieve better functional and cosmetic outcomes (3–6). The two-stage approach separated VC correction and reconstruction of the urethral plate from urethroplasty, simplifying proximal hypospadias repairs with a lower complications rate, and has become the preferred method for many surgeons (7, 8). The comparative studies of these two techniques indicate a higher complication rate of TPIFU (9, 10). During the past decades, researchers have modified the surgical technique of TPIFU in order to improve the success rate. Ferro et al. argued that cutting a wedge and a V-shaped suture at the ends of the flap could help prevent any circumferential suture line, avoiding proximal anastomotic stricture and distal meatal stenosis (11). Huang et al. modified the TPIFU by performing straightforward and reliable *in situ* tubularization of the flap, reducing urethral stricture incidence. Nevertheless, as one of the most common techniques for treating severe hypospadias, limited studies focus on the reoperation frequency after TPIFU.

In the present study, we reported our clinical outcomes of proximal hypospadias treated with one-staged TPIFU and analyzed the reoperation frequency. We hypothesized that although the complication rates after primary TPIFU were relatively high, the cure rate after two or three operations was acceptable.

## Materials and methods

### Patients

The clinical data of severe hypospadias patients who underwent primary TPIFU were retrospectively reviewed and analyzed from December 2018 to December 2019 in the department of Urology at Beijing Children's Hospital, National Center for Children's Health. For efficient reoperation frequency analysis, we identified that no complications occurred for 12 months after the date of last-time surgery as the standard of short-term cure. All patients achieved the standard of cure after primary surgery or accepted subsequent surgery handling with complications. The exclusion criteria included patients lost to follow-up or those with insufficient follow-up time (less than 12 months) after the last surgery. The study was conducted in accordance with the Declaration of Helsinki (as revised in 2013). This retrospective study was approved by the ethics committee of Beijing Children's Hospital, Capital Medical University, National Center for Children's Health [IEC-C-006-A04-V.06, (2022)-E-030-R]. Individual consent for this retrospective analysis was waived.

We used a stepwise approach to manage the curvature suggested by Castagnetti et al., and severe hypospadias was defined as the residual VC was higher than 30° after degloving (12). An orthopedic protractor measured the VC. As for the meatus position, we adopted the meatal position-based classification proposed by Duckett and was further simplified as distal (normal, fossa navicular, coronal sulcus, and distal shaft), mid-penile (middle shaft) and proximal (proximal shaft, penoscrotal, scrotum, and perineum) (13). Measurement of intrinsic parameters, suture materials, and strategies for urethroplasty were standardized between six surgeons with at least 15 years of experience in hypospadias surgery. Routine follow-up for all patients included assessment in the clinic at three months, then 12 months postoperatively, and continuing annual telephone interview or outpatient review. Urethroplasty complications included fistula, urethral stricture, and diverticulum. The urethral stricture was diagnosed by obstructive voiding symptoms, and cystoscopy was performed for further confirmation when managing urethral stricture. All the complications were managed by the original surgeon.

### Surgical techniques

**Figure 1:**
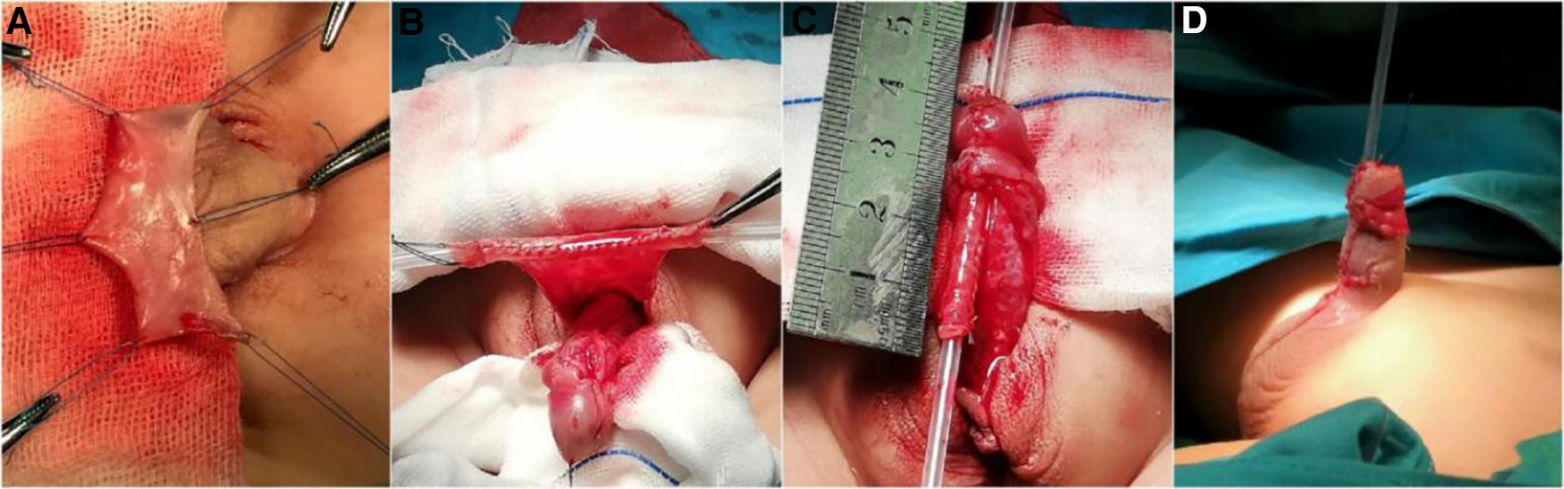
Surgical procedure of TPIFU (the Duckett’s procedure). (**A**) Separate the dorsal skin flaps. (**B**) Clip the rectangular flap from the inner prepuce, and roll the mobilized foreskin into a tube over a catheter. (**C**) Transpose the tubulate urethra ventrally through the glans channel and anastomose with the native urethra. (**D**) Postoperative appearance. TPIFU, transverse preputial island flap urethroplasty.

The TPIFU is the typical Duckett technique described in the previous literature (14). A stepwise approach was enforced during the operation: we fully degloved the penile shaft, freeing all ventral tissues, and radically dissected dartos. VC was evaluated with an artificial erection, and if the residual curvature less than 30°, dorsal plication was performed to correct it, we performed tubularized incised plate (TIP) urethroplasty or Onlay flap technique according to the UP condition. If the residual curvature was more than 30°, we transected the urethral plate at the corona, and dorsal plication was performed if the chordee was still present. For patients whose urethral plate was transected, we performed TPIFU. Release the urethral plate and urethra, release ventral fibrotic tissue, and drop back the meatus to the penoscrotal junction or the proximal shaft. Perform artificial erection by injecting normal saline into single corpora. The flap length is the distance from the retracted meatus to the glans tip after correcting VC. Subsequently, clip a 12-mm wide rectangular flap from the inner prepuce, and roll the mobilized foreskin into a tube over a catheter. Transpose the tubulate urethra ventrally through the glans channel and anastomose with the native urethra with 6-0 absorbed PDS. The relaxed vascularized and deepithelialized tissue was dissected to cover the neourethra. Then, the foreskin was sutured together to cover all of the penis. The surgical procedure is detailed in Figure 1. Finally, the penis body was bandaged with gauze and an elastic bandage.

### Statistical analysis

Patients' data were analyzed with R software (Version 4.0.3, http://www.r-project.org). The normal distribution and the homogeneity variance test were presented as mean ± SD, non-normal distribution or inhomogeneity variance test was presented as median and interquartile range. Continuous data were expressed as a percentage (%).

## Results

**Figure 2:**
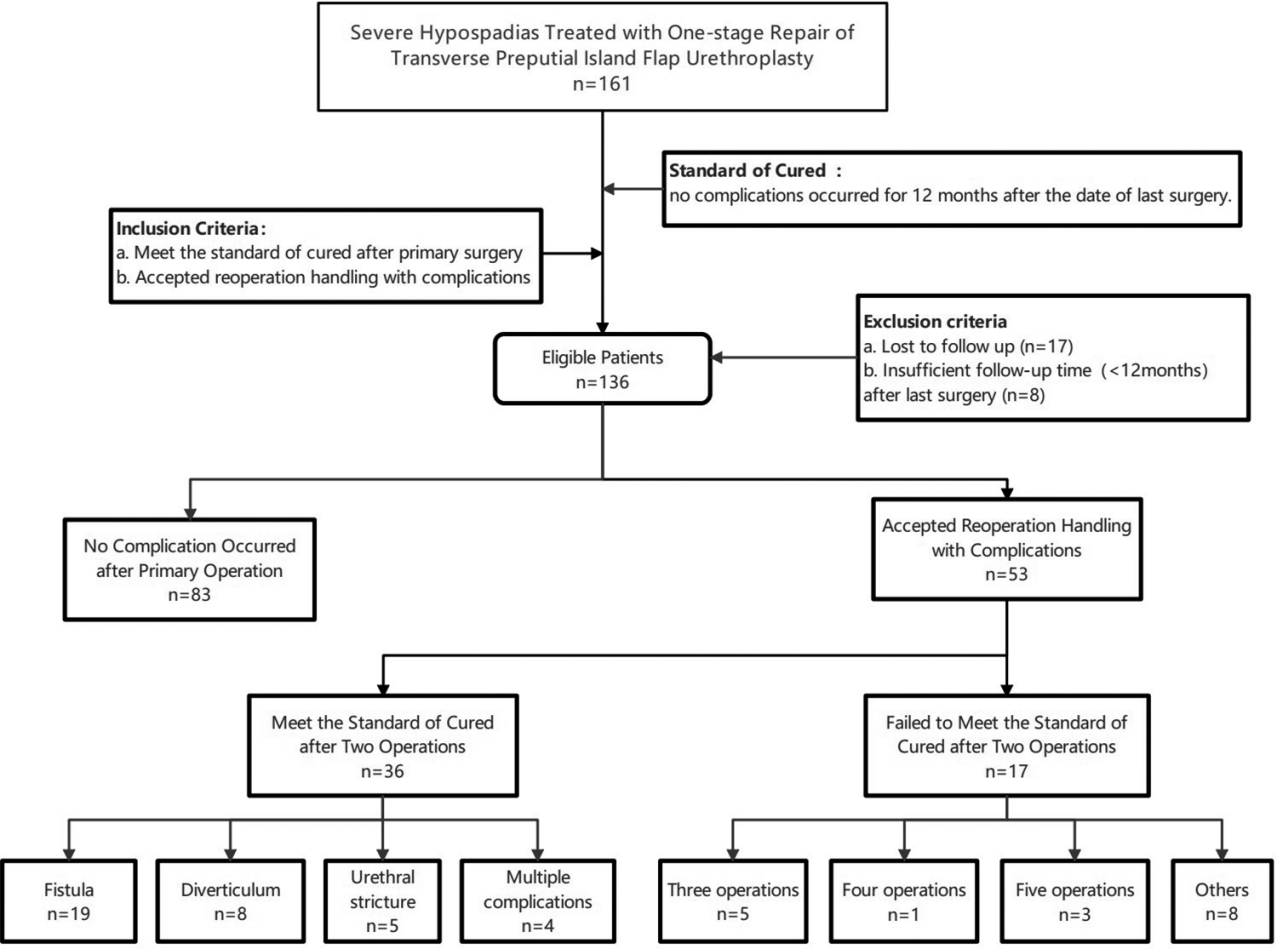
Reoperation analysis of TPIFU treating severe hypospadias. TPIFU, transverse preputial island flap urethroplasty.

Of 161 patients with severe hypospadias treated by TPIFU (The Duckett's Procedure) from December 2018 to December 2019 in the department of Urology at Beijing Children's Hospital, 136 met the criteria and were included in this study (Figure 2). The median age at primary surgery was 22.5 months (range from 13 to 132 months). All the patients were followed up for 34 months as the median time (range 22–50 months). Preoperative degree of VC is 60.0 [45.0, 80.0] °. Before VC correction, there were 44 cases of a urinary meatus in the middle penile, 56 cases of a urinary meatus in the proximal penile, 31 cases of a urinary meatus in the penoscrotal, and five cases of a urinary meatus in the perineum. After VC correction, 61 cases of urinary meatus in the proximal penile, 56 cases in the penoscrotal, and 19 cases of urinary meatus in the perineum. The median length of the deficient urethra was 3.5 cm (range 2.5 cm–6.0 cm). Intraoperative data of patients are summarized in Table 1.

**Table 1:** Characteristics of patients treated with TPIFU.

No. of patients	136
Age of surgery (months)	22.5 (19.0;28.2)
Length of penis (cm)	3.50 (3.00;4.00)
Hypoplastic urethra	93 (68.4%)
Ventral Curvature	
Preoperative degree (°)	60.0 (45.0;80.0)
Point of greatest bending	
Distal shaft	34 (25.0%)
Middle shaft	94 (69.1%)
Proximal shaft	8 (5.88%)
Correcting of VC	
Degloving	21 (15.44%)
+Urethra-plate transection	
Degloving	
+Urethra-plate transection	115 (84.56%)
+Dorsal plication	
Location of meatus before VC correction	
Middle penile	44 (32.4%)
Proximal penile	56 (41.2%)
Penoscrotal	31 (22.8%)
Perineum	5 (3.68%)
Location of meatus after VC correction:	
Proximal penile	61 (44.9%)
Penoscrotal	56 (41.2%)
Perineum	19 (14.0%)
Length of deficient urethra (cm)	3.50 [3.00;3.50]
**Preputial island flap**	
Length (cm)	3.50 [3.00;3.50]
Width (cm)	1.20
Smoothness	
Smooth	81 (59.6%)
Ordinary	42 (30.9%)
Uneven	13 (9.56%)
Elasticity	
Good	94 (69.1%)
Normal	38 (27.9%)
Bad	4 (2.94%)

TPIFU, transverse preputial island flap urethroplasty; VC, ventral curvature.

As shown in Figure 2, a total of 83 (61%) patients met the standard of cure, which means no complication was identified for at least 12 months after the primary surgery. Among the other 53 (39%) patients who accepted reoperation handling with complications, 36 (26%) patients met the standard of cured after two operations (reoperated once). Among them, fistulas developed in 19 patients who underwent fistula repair. Five urethral strictures were treated successfully with one dilatation, and eight patients concurrent with diverticula were treated with resection of redundant tissue and multilayer closure. Besides, there were four patients with multiple complications handled by one reoperation. Two patients combined fistula and diverticula underwent fistula repair plus diverticula resection, and the other two patients underwent diverticula resection plus urethroplasty due to the combination of urethral stricture and diverticula. Five (4%) patients were cured after three operations (reoperated twice). Two of them accepted extra dilatation twice because of urethral stricture. Two patients successively underwent dilatation and fistula repair. One was reoperated twice due to urethral stricture and diverticula. At the date of the last follow-up, 17 (12.5%) cases who had accepted more than two surgeries did not achieve the cured standard yet. One child had repeated stricture and had been dilated three times after the initial surgery. Three cases remain with complications after five-time surgical treatments. One was dilated four times (still with diverticula), one was dilated three times and eventually developed into multiple complications including urethral stricture, fistula, diverticula, urethral stone, and recurrent curvature, underwent open surgical reconstruction. One sequentially accepted salvage urethrotomy and urethral reconstruction after two dilatations due to the stricture. Other eight patients accompanied with complications after two operations had delayed their further surgical treatment for myriad reasons.

Table 2 summarizes the reoperation data of TPIFU. After primary surgery, 24 patients (17.6%) developed fistula, 17 patients (12.5%) developed urethral stricture and 11 patients (8.1%) developed diverticulum. 17 patients did not meet the standard of cure after the second surgery, of which five patients remained fistula, five patients remained urethral stricture, and seven patients remained diverticulum. Overall, 61% (85 patients) of the patients met the standard of cured after primary operation in the present study, with a once-reoperation (two operations) cure rate was 87.5% (119 patients). 91.2% (124 patients) were cured in three operations.

**Table 2:** Operation times of TPIFU.

Complications after:	Primary operation	Second operation	Third operation
Fistula	24/136	5/17	2/4
Urethral stricture	17/136	5/17^a^	2/4
Diverticulum	11/136	7/17	2/4
No. Operation	**One**	**Two**	**Three**
No. Cured Cases (%)	83 (61.0)	119 (87.5)	124 (91.2)

^a^ One patient sequentially accepted urethrostomy and fistula reparation after two dilatations due to the stricture. He was not included in the analysis of complications after third operation. TPIFU, transverse preputial island flap urethroplasty.

## Discussion

Consistently, there is a lack of a clear definition of severe hypospadias. The clinically accepted preoperative meatal position to classify the severity of hypospadias but the meatal position would change after VC correction. Therefore, a definite classification can only be completed at intra-operative findings. The curvature and meatal position were both important factors to describe the severity of hypospadias (15). In 2010, Castagnetti et al. (16) performed a systematic review of the management of primary severe hypospadias, and suggested 30° as the minimum cut-off point for defining a curvature as clinically significant. A 1999 survey of members of the American Academy of Paediatrics noted that 30° curvature was the threshold at which surgeons almost invariably agreed that non-operative management was no longer an option (17). A global survey of paediatric urologists, paediatric surgeons and plastic surgeons, published in 2011, broadly confirmed these results (18). For patients with residual VC was higher than 30° after degloving, transection of the urethral plate is inevitable, and grafts such as buccal mucosa graft and flaps have been used to achieve full correction of the penile curvature (19). In the present study, we used a stepwise approach to manage the curvature, and severe hypospadias was defined as the residual VC was higher than 30° after degloving. Of 136 patients who underwent TPIFU, the mean degree of VC was 60 °. The urethral plate was excised in all the cases, and dorsal plication was performed in 115 (84.56%) patients.

Although surgeons have been making various efforts or modifications to optimize the procedure, managing severe hypospadias is challenging, and the best options for fewer complications are still debated. Notwithstanding the dispute on single-stage vs. two-stage repairs is ardent, the purpose of the operation was to provide a tension-free, well-vascularized tubularized neourethra and improve postoperative wound healing (20). Recently, a new three-stage approach have been proposed to offer an alternative option for patients who presenting with diffuse scarring of the urethral plate and a shortage of penile skin for closure. It is believed that the selection of surgical techniques in hypospadias repair is highly based on the patient's anomaly characteristics and the surgeon's preferences. TPIFU is one of the most common techniques for severe hypospadias. Although researchers have made modifications to improve prognosis, the satisfactory complications rate has not been reached (21–23). Indeed, the complication rates of TPIFU, ranging from 13% to 91%, vary widely in the literature and across countries (24). We reviewed the literature and summarised the outcomes of articles published on proximal hypospadias treated with TPIFU, as shown in Table 3 (14, 20, 23, 25–37).

**Table 3:** Literature review of one-stage TPIFU (14, 20, 23, 25–37).

Author; Year	No. Patients	Age (m)	Follow up (m)	No. Complications	Fistula	Urethral stricture	Diverticulum	Wound dehiscence	Others
Castañón, E, 2000 (25)	42	37 (12–108)	/	16	9	3	2	/	2
Chuang, 1995 (26)	103	47 (5–168	/	31	25	5	1	/	/
Cui, 2020 (27)	155	21.6 (6–144)	62.4 (3–120)	84	49	26	9	/	/
Daboos, 2020 (23)	160	54	36	28	12	12	/	4	/
Dewan, 1991 (28)	189	6–120	25.1 ± 23.7	93	65	55	5	/	/
Ghali, 1999 (29)	148	38.4 (18–144)	23 (6–53)	48	22	30	7	/	3
Han, 2020 (30)	161	14–186	13.7 (12–17)	42	25	11	5	/	/
Hayashi, 2001 (31)	13	23 (10–36)	36 (30–44)	1	1	/	/	/	/
Huang, 2017 (20)	32	11 (7–23)	23 (12–38)	6	6	/	/	/	/
Huang, 2017 (32)	65	33.6 (7–144	19 (14–30)	10	10	0	/	/	/
Lyu, 2019 (33)	40	26.2 ± 13.8	12	19	14	4	1	/	/
Macgillivray, 2002 (34)	24	27.5 (21–35)	62.5 (4–100)	10	9	1	/	/	/
Patel, 2004 (35)	14	16.8 (8–74)	170 (144–253)	3	2	1	/	/	/
Sorber, 1997 (36)	70	(6–168)	16.7 (12–92)	16	6	4	/	4	2
Wang, 2019 (14)	320	15.1 (11–64)	40.2 (1–156)	125	53	31	41	/	/
Zheng, 2012 (37)	25	145.4 ± 128.4	38.7 (22–60)	6	4	4	/	1	/

TPIFU, transverse preputial island flap urethroplasty.

**Figure 3:**
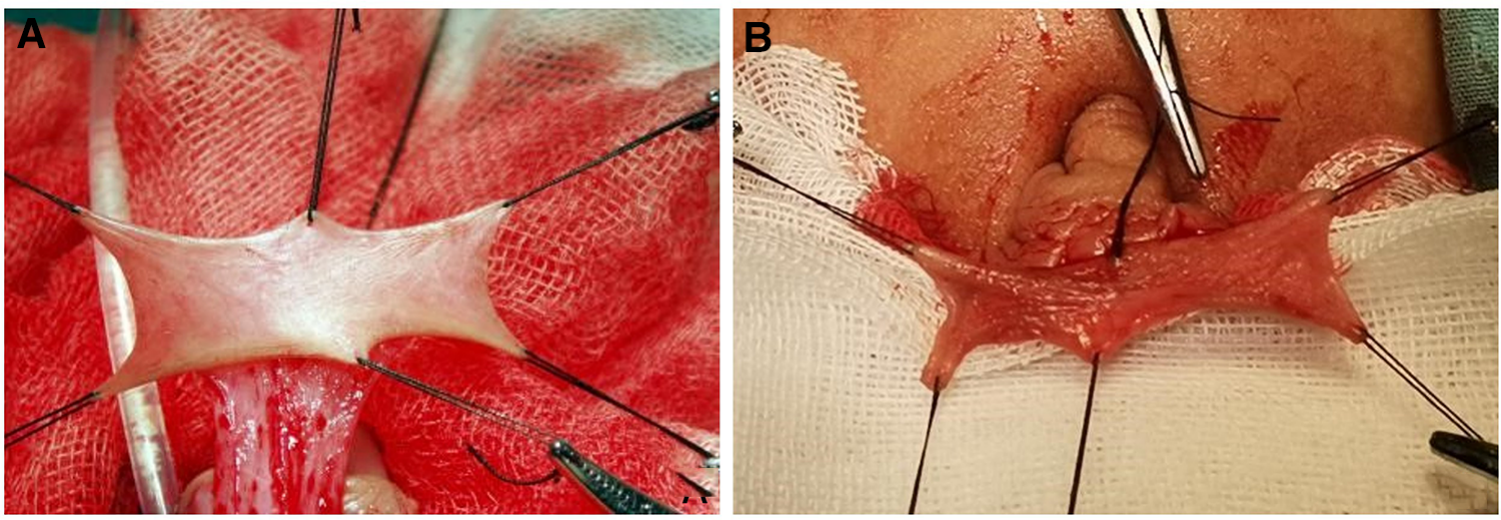
Preputial island flap condition. (**A**) In good condition, the flap is smooth and presents good elasticity. (**B**) In bad condition, the flap is stiff and not smooth.

In the present study, complications were defined as any complaints that required surgical correction, including fistula, urethral stricture, and diverticulum. The complication rate of primary operation was 39%, with fistula (24 patients, 17.6%) being the most common complication, similar to the previous studies (38). Both fistula and urethral stricture were related to the poor blood supply to the neourethra and the native urethra (39). Cui et al. suggested that surgeons should protect the vascular pedicle of the inner sheath of the foreskin, ensuring the transverse flap is as thick as possible so that the vascular tissue can be preserved. The marginal skin with poor blood supply at the end of the neourethra must be removed when the neourethra and the native urethra are anastomosed. Compared with the fistula, the urethral stricture is a more distressing complication from the patient's perspective. The main cause of strictures is the circular scar between the native urethra and the flap, and it may require salvage urethrotomy and even urethral reconstruction (40). In the present study, 17 patients (12.5%) developed urethral stricture, of which six underwent two or more dilatations, and two underwent open surgical reconstruction. As the urethral stricture is difficult to deal with, researchers have modified the surgical technique (11). In our experience, in addition to protecting the blood supply, a tension-free anastomosis between the neourethra and the native urethra is the key point to decreasing the incidence of urethral stricture. An oblique anastomosis should be adopted between the neourethra and the native urethra to increase the area of the anastomotic surface and the blood supply of the anastomosis. For patients developed urethral stricture, we usually choose the appropriate treatment according to the patient's urinary symptoms. For patients with less severe symptoms, dilatation was chosen in preference. For patients with more severe symptoms after repeated dilatations, we will perform salvage urethrotomy and urethral reconstruction. As for the diverticulum after TPIFU, it was hypothesized that the flap's lack of attachment to the surrounding tissues makes the neourethra more elastic and thus prone to progressive dilation (41). On the other hand, some researchers believed that the common cause of diverticulum is that tube is flail between only two fixed points at the hypospadic and the new meatus. In the present study, 12 (9.1%) patients concurrent with diverticula were treated with resection of redundant tissue and multilayer closure. Based on our clinical practice, the incidence of diverticulum was related to the smoothness and elasticity of the preputial island flap (As shown in Figure 3). Two-stage technique was recommanded for those preputial island flap condition is poor.

To the best of our knowledge, this is the first study to focus on the reoperation frequency after TPIFU. Although the TPIFU developed relatively high complication and reoperation rate after the primary surgery, the result may be different if we focus on the surgery number to cure the patients. In the present study, 83 (61%) patients achieved short-term cured through a single operation, which means more than 50% of patients could be free from the second procedure. On the other hand, TPIFU provides the advantages of minimizing operative trauma, allowing the use of unscarred skin with good vascularity and decreasing the number of hospitalizations, decreasing the infection risk and caregiving burden for parents. As we gain an improved understanding of the interplay of psychological factors of children, including timing in genital awareness, sexual orientation, and body image, more procedures may lead children and parents to develop lower opinions on the repair of hypospadias and worse sexual and psychological results (42). As the National Center for Children's Health in China, our center has more patient resources and a well-structured educational system, helping doctors overcome the steep learning curve of TPIFU. Snodgrass et al. suggested that to gain expertise from greater volumes of cases, surgeons/teams should be designated for proximal hypospadias repair in each center (43). Castagnetti et al. argued that surgeon experience is more important than technique (15). When choosing the type of repair, personal preference, training, and success rate when using a specific technique should be considered as important variables. Every surgeon should develop their own strategy and focus on mastering only a few techniques.

The present study has several limitations, including the retrospective nature, a small number of patients, and short-term follow-up. The long-term complications such as curvature recurrence and erectile pain did not analyze due to insufficient follow-up duration. Besides, we did not measure uroflow and postvoid residual volume in all patients. Obstructive urinary flow pattern seems common in patients who underwent proximal hypospadias repair, which may remit spontaneously with age. Thus, we believe conservative treatment with watchful observation may avoid unnecessary intervention. Further multicenter studies with larger sample sizes are required. Besides, we will publish a follow-up series to further prospective validate and extend the result of the present study (Trial registration number ChiCTR1900023055).

## Conclusions

To the best of our knowledge, this is the first study to focus on the reoperation frequency after TPIFU. Although the complication rates after primary TPIFU were relatively high, the cure rate after two or three operations was acceptable. Multi-institutional work with a large number of cases and long-term follow-up is needed in the future.

## Data Availability

The datasets used and/or analysed during the current study are available from the corresponding author upon reasonable request.
